# The Potential Association of Delayed T Lymphocyte Reconstitution Within Six Months Post-Transplantation With the Risk of Cytomegalovirus Retinitis in Severe Aplastic Anemia Recipients

**DOI:** 10.3389/fcimb.2022.900154

**Published:** 2022-05-25

**Authors:** Wenjian Mo, Xiangting Chen, Xu Zhang, Shunqing Wang, Ling Li, Yuehong Zhang

**Affiliations:** ^1^ Department of Hematology, Guangzhou First People’s Hospital, School of Medicine, South China University of Technology, Guangzhou, China; ^2^ Department of Ophthalmology, Guangzhou First People’s Hospital, School of Medicine, South China University of Technology, Guangzhou, China; ^3^ Department of Biology, University of North Dakota, Grand Forks, ND, United States

**Keywords:** cytomegalovirus infection, hematopoietic stem cell transplantation, severe aplastic anemia, immune reconstitution, lymphocyte subsets

## Abstract

**Background:**

Delayed immune reconstitution after allogeneic hematopoietic stem cell transplantation (HSCT) is significantly associated with cytomegalovirus (CMV) infection. The aim of this study was to observe the recovery trend of peripheral lymphocyte subsets and immunoglobulins in HSCT recipients who developed CMV retinitis (CMVR).

**Methods:**

We identified 37 CMVR cases and 303 non-CMVR controls in this case-control study from a database of 404 consecutive severe aplastic anemia patients who received allogeneic HSCT at a single center between 2015 and 2020. We analyzed the transplant outcomes and immune reconstitution principles with a focus on lymphocyte CD series and immunoglobulin series within the first year post-HSCT.

**Results:**

Thirty-seven patients (55 eyes) were diagnosed with CMVR, with a mean onset time of 155 days post-HSCT. Among the 37 patients, one never had CMV detected in his blood but had a high CMV load in his intraocular fluid at the time of CMVR diagnosis. In the controls, 195 had CMV viremia and 108 did not. Compared with controls, CMVR cases had a longer duration of CMV viremia and a higher peak number of CMV load. T lymphocyte subsets including CD3, CD4 and CD8 were significantly lower in CMVR cases within six months after HSCT (all *p* < 0.05). Immunoglobulins also showed a slower recovery trend in CMVR cases. The recovery of B lymphocytes and natural killer cells exhibited no significant differences between the two groups.

**Conclusions:**

It is not enough to develop fundus screening strategies by merely relying on the CMV serostatus of recipients. Dynamic and continuous monitoring of T lymphocyte subsets, especially within six months post-HSCT, as well as serum immunoglobulin levels, can provide assistance with screening program of CMVR in HSCT recipients with severe aplastic anemia.

## Introduction

Opportunistic infections are non-negligible in transplant patients whose immune systems are artificially suppressed to minimize rejection. Cytomegalovirus (CMV) infection is one of the most common infectious complications among hematopoietic stem cell transplant (HSCT) recipients and remains a significant cause of morbidity and mortality (Boeckh et al., 2003; Ljungman et al., 2019). The speed of immune reconstitution post-HSCT is closely related to the survival rate, infection risks and graft failure. However, many factors may affect the immune reconstitution post-HSCT such as the primary disease, graft function, donor characteristics, conditioning regimen, and graft versus host disease (GVHD) and so on (van den Brink et al., 2015; Bhatt and Bednarski, 2020). Severe aplastic anemia (SAA) is an immunologically mediated disorder characterized by hypocellular bone marrow and pancytopenia, and HSCT is a recommended curative option for SAA (ElGohary et al., 2020). Unlike transplant recipients with other malignant hematological diseases, many SAA patients have experienced failure of immunosuppressive therapy before HSCT, in addition to the routine use of immunosuppressants to prevent GVHD and graft failure in the peri-transplant period. Nearly all SAA patients use anti-thymocyte globulin (ATG), a polyclonal anti-lymphocyte antibody, as an induction drug for HSCT, although it has been clearly demonstrated that ATG can increase the risk of CMV infection (Wu et al., 2017; Johnsrud et al., 2020; Kang et al., 2021). Moreover, SAA patients also face a problem of poor hematopoietic microenvironment, which may affect donor-derived immune cell development (Chao et al., 2010; Wu et al., 2015). All of the above significantly delay immune reconstitution post-HSCT, so these unique features in SAA HSCT recipients may also contribute to the development of an active CMV infection.

CMV retinitis (CMVR), compared to other CMV end-stage organ diseases, is a more localized and late-onset HSCT-related complication; it behaves according to unique immune reconstitution rules (Hiwarkar et al., 2014; Meng et al., 2020). Without treatment, CMVR may cause irreversible visual impairment due to its aggressive feature. Although active fundus screening based on symptoms and positive CMV infection monitoring can improve the early diagnosis of CMVR, it should be noted that some patients have turned CMV-seronegative after systemic antiviral therapy at the time of CMVR diagnosis (Jeon and Lee, 2015; Zhang et al., 2017; Vassallo et al., 2020). Patients may ignore advice regarding regular ocular examinations due to the deceptiveness of early asymptomatic CMVR and the duration of CMV-seronegative status after HSCT. This means that it is not enough to put in place a strategy for CMVR screening that depends solely on the recipient’s CMV serum status.

We hypothesized that the immune monitoring data of recipients could improve CMVR risk assessment and assist in CMVR screening and surveillance. Therefore, we designed this retrospective study to summarize the principles of immune reconstitution in patients with CMVR after transplantation. In our medical institution, all HSCT recipients undergo routinely immune monitoring for at least one year after transplantation. Therefore, the identification of immune predictors would not impose additional financial burdens on patients while providing further means, in addition to positive CMV serostatus, for the screening of early asymptomatic CMVR.

## Methods

### Study Design and Patients

This retrospective case-control study targeted consecutive SAA patients who received allogeneic HSCT at Guangzhou First People’s Hospital in China between January 2015 and December 2020. Patients were divided into two groups, and all CMVR patients were included in the case group. CMVR was diagnosed by an experienced ophthalmologist and documented by retinal photography. When the diagnosis of CMVR was uncertain, the CMV DNA viral load of aqueous humor was evaluated to assist diagnosis. Non-CMVR patients were included in the control group which should follow three exclusion criteria. First, patients who died within two years after HSCT were excluded. The main consideration for excluding these patients was that CMVR could not be excluded without more than a one-year follow-up. Second, patients who had no complete clinical data due to an initial failure of transplantation, a lapse in follow-up or other reasons were excluded. Finally, patients whose retinitis was induced by other pathogens, such as the herpes simplex virus, varicella-zoster virus, or Epstein-Barr virus (EBV), which might cause ocular manifestations similar to CMV, were excluded. The collected data included demographic and clinical data, virological findings, and immunological variables. This study was approved by the institutional review board of Guangzhou First People’s Hospital (IRB B-2022-005-01) and was conducted in accordance with the Helsinki Declaration.

### Virus Detection and Assessment Criteria

Before HSCT, all recipients underwent serological tests for CMV, herpes simplex virus, varicella-zoster virus, EBV, and antibodies to hepatitis B and C virus. After HSCT, CMV-DNA and EBV-DNA were tested at least weekly for the first three months, biweekly from the fourth to the sixth month, and monthly from the seventh to the twelfth month. Immunoglobulin (Ig) G and IgM for the herpes simplex virus were tested monthly for the first three months after transplantation. The threshold for CMV viremia was met if CMV-DNA was ≥ 500 copies/mL in two consecutive measurements in our medical institution. The start time of CMV infection was defined as the first CMV-DNA ≥ 500 copies/mL, and the end time of CMV infection was defined as testing negative in two consecutive measurements. EBV viremia was defined if EBV-DNA was ≥1000 copies/mL in two consecutive measurements. EBV disease was defined as either probable EBV disease (significant lymphadenopathy or other end-organ diseases accompanied by a high EBV load) or proven diseases (histologically confirmed EBV positive post transplantation lymphproliferative disorders or other end-organ diseases) (van Esser et al., 2002; van der Velden et al., 2013). Patients with EBV infection were given the preemptive therapy of weekly infusion of rituximab (375 mg/m^2^) until the EBV-DNA <1,000 copies/mL. Patients with EBV disease were given four times weekly infusion of rituximab. All HSCT patients were supplemented by intravenous IgG at a dose of 0.4 g/kg on days 1, 11 and 21 post-HSCT and patients who received rituximab needed to be supplemented additional IgG at a dose of 0.4 g/kg once every two weeks, four times in total. The prevention and treatment protocols for EBV infection based on rituximab administration in two cohorts were the same.

### Ophthalmological Examination

Before transplantation, all patients were scheduled for ophthalmic examination to exclude potential ocular infection. After transplantation, a fundus screening for CMVR was mainly performed in patients with visual symptoms, such as blurred vision or floater scotoma, and in patients with CMV viremia. Patients with visual abnormalities were arranged to undergo an ophthalmic examination as soon as possible. For patients with CMV infection, regular fundus screening was performed every other week within half a year and monthly 6 to 12 months after HSCT. The ophthalmic examination included the best-corrected visual acuity, slit-lamp biomicroscopy, intraocular pressure measurements, dilated fundus examination, and color fundus photography. Samples of intraocular fluid from the anterior chamber were collected from confirmed or suspected CMVR patients to quantify CMV DNA loads.

### Immune Reconstitution Monitoring

In our medical institution, transplant recipients were hospitalized until they were determined to be clinically stable. Most laboratory values used to assess immune reconstitution and infection were measured twice weekly during inpatient care and at least weekly during outpatient encounters as part of routine clinical care. The laboratory data collected in this study included lymphocyte CD series and Ig series. The absolute numbers of total lymphocytes, T lymphocytes (CD3, CD4 and CD8), B lymphocytes (CD19), and natural killer cells in the peripheral blood, as well as the serum levels of IgA, IgE, IgG, and IgM, were counted by flow cytometry (Becton Dickinson Biosciences, San Jose, CA, USA) at 1, 2, 3, 6 and 12 months after HSCT.

### Statistical Analyses

The statistical analysis was performed using R software (Version 4.0.4). Continuous variables were expressed as median and range, and categorical data were presented as frequency and percentage (%). Comparisons between two groups were performed using *t*-tests and Wilcoxon tests for continuous variables and chi-square tests for categorical variables. Correlation between the lymphocytes was performed with Pearson correlation analysis. Linear regression and mixed effect linear regression model were used to explore the association of each parameter with CMVR. *P* values less than 0.05 were considered statistically significant.

## Results

### Patient Characteristics

We documented 410 episodes of allogeneic HSCT in 404 SAA patients from January 2015 to December 2020 at Guangzhou First People’s Hospital in China. Fifty-three patients without CMVR were excluded due to death within two years, a lapse in follow-up or other reasons. Six patients were excluded due to a failure of initial transplantation. Five patients with acute retinal necrosis were also excluded. Finally, the remaining 37 CMVR patients and 303 non-CMVR controls meeting all the criteria mentioned above were included in this study and analyzed. The general characteristics of the study population are summarized in **Table 1**. The following characteristics had no difference between the two groups: recipient age and sex, interval from SAA diagnosis to HSCT, failure of prior immunosuppressive therapy, history of heavy transfusion, donor female sex, infusion cell dose, varicella-zoster virus infection, hepatitis B and C virus infection, GVHD, basiliximab therapy, and conditioning regimens (all *p* > 0.05). The exception to this was that EBV infection, rituximab therapy and donors showed significant differences between the two groups. Compared with controls, CMVR cases had higher proportions of EBV co-infection (*p* < 0.0001), rituximab therapy (*p* < 0.0001) and alternative donors (*p* = 0.001).

**Table 1:** General characteristics of the study population.

Characteristic	CMVR cases (n = 37)	Controls (n = 303)	*p* value
Recipient age-yr	28 (6–51)	27 (6–58)	0.183^∧^
Recipient female sex, n (%)	12 (32.4)	141 (46.5)	0.104^#^
Interval from SAA diagnosis to HSCT (mth, range)	11 (1–240)	3 (1–240)	0.209^∧^
Failure of prior immunosuppressive therapy, n (%)	6 (16.2)	73 (24.1)	0.459^#^
History of heavy transfusion, n (%)	21 (56.7)	121 (39.9)	0.051^#^
Donor female sex, n (%)	14 (37.8)	117 (38.6)	0.927^#^
Infusion cell dose			
mononuclear cell (×10^8^/kg), median (range)	11.1 (8.9–12.9)	5.4 (1.9–12.3)	0.665^∧^
CD34 cells (×10^6^/kg), median (range)	10.9 (6.7–16.8)	4.6 (1.7–10.1)	0.571^∧^
EBV infection, n (%)	19 (51.4)	47 (15.5)	<0.0001^#^
Varicella-zoster virus infection, n (%)	6 (16.2)	43 (14.2)	0.741^#^
Hepatitis B and C virus infection, n (%)	8 (21.6)	56 (18.5)	0.645^#^
Grade 3 and 4 acute GVHD, n (%)	6 (16.2)	72 (23.7)	0.312^#^
Chronic GVHD, n (%)	7 (18.9)	39 (22.8)	0.310^#^
Rituximab therapy, n (%)	17 (46.0)	17 (5.6)	<0.0001^#^
Basiliximab therapy, n (%)	2 (5.4)	10 (3.3)	0.512^#^
Donor type, n (%)			
MSD	3 (8.1)	105 (34.7)	0.001^#^
Alternative donor (HID and URD)	34 (91.9)	198 (65.3)	
Conditioning regimens, n (%)			
CY + ATG	0 (0)	20 (6.6)	0.257^#^
FCA	15 (40.5)	89 (29.4)	
BU + CY + ATG	15 (40.5)	140 (46.2)	
PTCy + ATG	7 (19.0)	54 (17.8)	

CMVR, cytomegalovirus retinitis; HSCT, hematopoietic stem cell transplantation; GVHD, graft-versus-host disease; MSD, human leukocyte antigen-matched sibling donor; HID, haploidentical donor; URD, unrelated donor. The following conditioning regimens were administered to the patients in our study: cyclophosphamide at 50 mg/kg/day for four days and anti-thymocyte globulin at 2.5 mg/kg/day for four days (CY + ATG); fludarabine at 30 mg/m2/day for four days, cyclophosphamide at 40 mg/kg/day for four days and anti-thymocyte globulin at 2.5 mg/kg/day for four days (FCA); busulfan at 3.2 mg/kg/day for two days, cyclophosphamide at 50 mg/kg/day for four days and anti-thymocyte globulin at 2.5 mg/kg/day for four days (BU + CY + ATG), fludarabine at 30 mg/m2/day for four days, cyclophosphamide at 40 mg/kg/day for four days (two days before transplant and two days post-transplant) and anti-thymocyte globulin at 2 mg/kg/day for three days (PTCy + ATG). For MSD HSCT patients, CY + ATG and FCA conditionings were applied. For URD HSCT patients, FCA conditioning was applied. For HID HSCT patients, BU + CY + ATG and PTCy + ATG conditioning were applied. ^∧^T-test; ^#^chi-square.

### Clinical Manifestations of CMVR Patients

Thirty-seven patients (55 eyes) were diagnosed with CMVR at a median of 155 days (range 22–339 days) following HSCT. Nineteen patients suffered unilateral CMVR and 18 had bilaterally involvement. All but one patient who developed CMVR at 22 days after transplantation, other patients developed CMVR three months later. CMV viremia following the HSCT was found in 36 CMVR patients (97.3%) and 19 (51.4%) suffered simultaneously EB viremia before the diagnosis of CMVR. PCR results for CMV and EBV in aqueous humor showed that 48 eyes (87.27%) had positive CMV. One CMVR patient had never had CMV detected in his blood but had a high CMV load in his aqueous humor at the time of diagnosis of CMVR. After combined intravitreal and intravenous injection of ganciclovir, his bilateral lesions subsided and visual acuity recovered. No intraocular EBV was detected in any case. Three main fundus presentations of CMVR were all present in our cases including the granular form, hemorrhagic/edematous form and frosted branch angiitis form. **Table 2** shows the ocular manifestations and clinical outcomes of these 37 CMVR patients.

**Table 2:** Ocular manifestations and clinical outcomes of 37 patients with CMVR.

Characteristic	According to patients (n = 37)	According to eyes (n = 55)
Gender		
Female	12 (32.4%)	
Male	25 (67.6%)	
Diagnosis		
SAA	27 (72.9%)	
vSAA	10 (27.1%)	
Involved eye		
OD	12 (32.4%)	30 (54.5%)
OS	7 (18.9%)	25 (45.5%)
OU	18 (18.6%)	
^*^Size of retinal lesions		
< 10%		24 (43.6%)
10% to 50%		22 (40.0%)
> 50%		9 (16.4%)
Treatment of CMVR		
Combined IVTG and systemic administration of antiviral drugs	28 (75.7%)	40 (72.8%)
IVTG only	2 (5.4%)	3 (5.4%)
CMV-CTL immunotherapy targeting drug-resistant CMVR	2 (5.4%)	3 (5.4%)
No treatment	5 (13.5%)	9 (16.4%)
^∧^Visual prognosis		
Improvement		27 (49.1%)
Stabilization		17 (30.9%)
Deterioration		11 (20.0%)
Follow up period-mth	18.2 (5.9–33.8)	
^#^One-year mortality	6 (16.2%)	

CMVR, cytomegalovirus retinitis; SAA, severe aplastic anemia; vSAA, very severe aplastic anemia; OD, right eye; OS, left eye; OU, both eyes; IVTG, intravitreal injection of ganciclovir; CMV-CTL, CMV specific cytotoxic T lymphocytes. ^*^The sizes of retinal lesions were classified as involving < 10%, 10% to 50% and > 50% of the total retinal area based on the fundus photograph. ^∧^The visual outcome was defined as improvement, stabilization and deterioration. Improvement was defined if best-corrected visual acuity was increased two or more lines. Stabilization was defined if the changed best-corrected visual acuity was less than two lines. Deterioration was defined as a decreased best-corrected visual acuity more than two lines. A Snellen chart was used to record the visual acuity data. ^#^The causes of death of the six patients were CMV pneumonia (2 cases), Epstein-Barr virus-related post-transplant lymphoproliferative disease (1 case), septic shock (1 case), graft rejection (1 case) and commit suicide (1 case).

### Comparison of Virological Findings Between Two Cohorts

**Figure 1:**
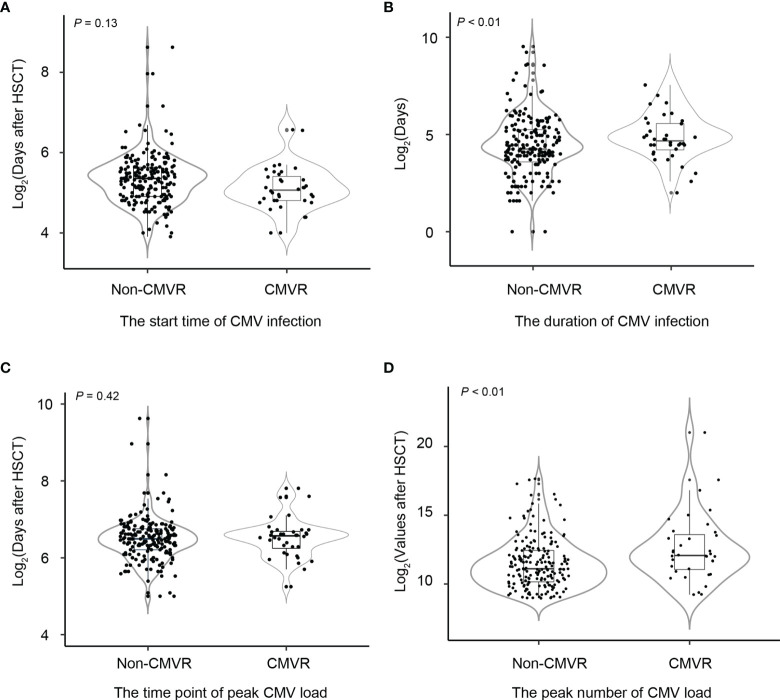
Comparisons of the start and duration time of cytomegalovirus (CMV) infection, the peak number of CMV copies, and the time point of peak CMV load between non-CMV retinitis (CMVR) patients and CMVR patients in severe aplastic anemia patients after hematopoietic stem cell transplantation **(A–D)**. Non-CMVR in this figure referred to patients with CMV infection but no retinitis (n = 195). The number of CMVR patients was 37.

Among 37 CMVR cases, there were 36 patients had CMV viremia. Of the 303 controls, 195 had CMV viremia and 108 did not. **Figure 1** shows the median start and duration of CMV infection, as well as the peak number of CMV load in the blood and the time point of peak CMV load after HSCT. The start time for CMV infection was comparable (a median of 33 days versus 41 days, *p* = 0.13) between the two groups. The infection duration was longer (a median of 112 days versus 19 days, *p* < 0.01) in cases than controls. The time point of peak CMV load had no difference (a median of 48 days versus 45 days, *p* = 0.42), but the peak number of CMV load in CMVR cases was higher than controls (a median of 4.29 versus 2.34× 10^4^ copies/mL, *p* < 0.01).

### Comparison of Immunological Findings Between Two Cohorts

**Figure 2:**
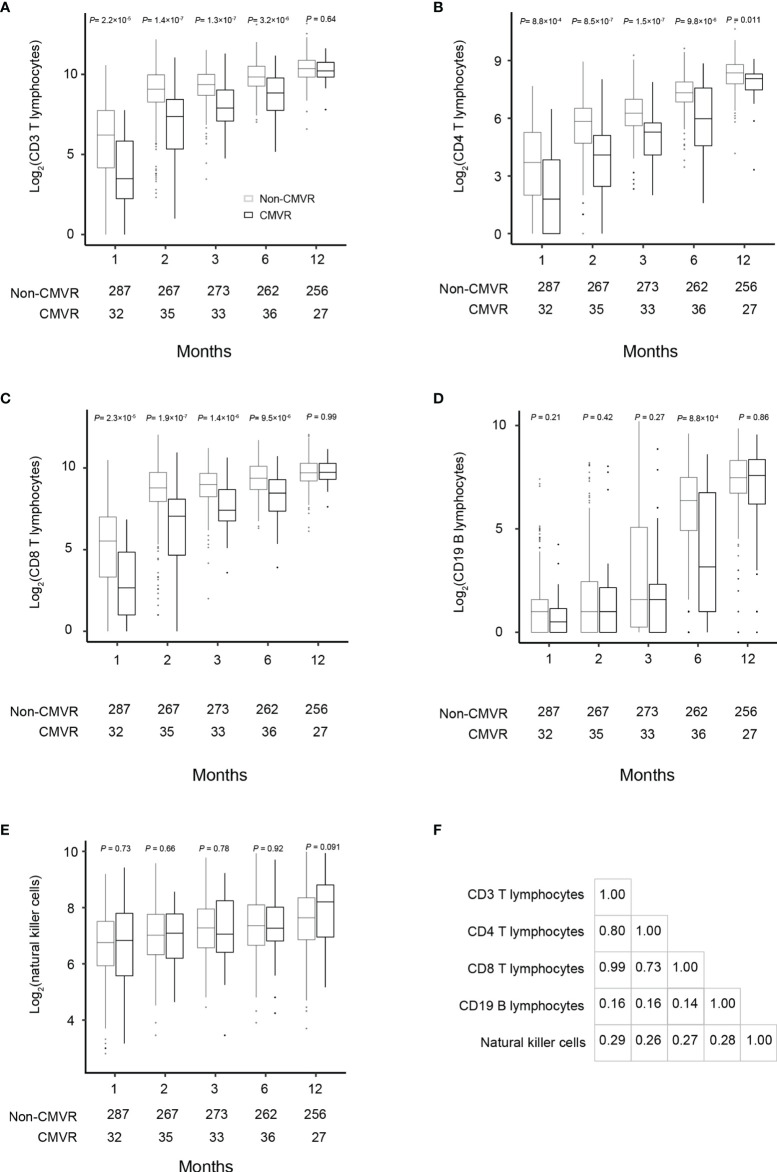
Comparisons of the total lymphocyte, CD3, CD4, CD8, CD19 and natural killer cell counts at 1, 2, 3, 6, and 12 months after hematopoietic stem cell transplantation between the CMVR patients and those who did not develop CMVR **(A–E)**. The number of samples used in each time point was indicated in each figure. Based on the fact that most CMVR occurred from the third month after transplantation, Pearson correlation was used to explore the correlation between lymphocytes. The data were assessed by the Pearson correlation coefficient indicated a significant correlation between CD3, CD4 and CD8 in the third month after transplantation **(F)**.

**Figure 3:**
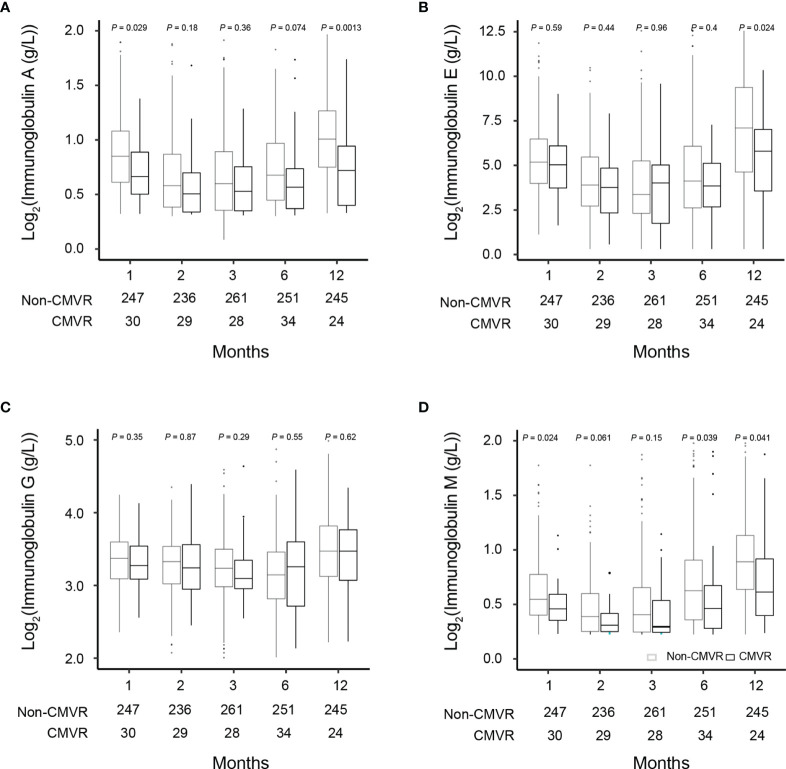
Comparisons of the immunoglobulin (Ig) levels in severe aplastic anemia patients who did and did not develop CMVR at 1, 2, 3, 6, and 12 months after hematopoietic stem cell transplantation **(A–D)**. The number of samples used in each time point was indicated in each figure.

**Figure 4:**
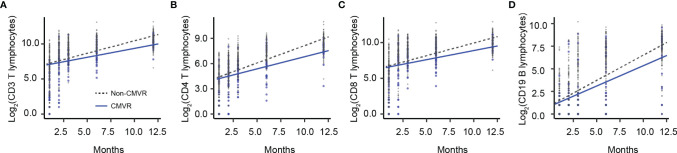
The overall trend of immune recovery of four T lymphocytes within one year after hematopoietic stem cell transplantation. **(A)** CD3 T lymphocytes, **(B)** CD4 T lymphocytes, **(C)** CD8 T lymphocytes, and **(D)** CD19 B lymphocytes.

T lymphocytes CD3, CD4 and CD8 were lower in CMVR cases than in controls during the first six months after HSCT (*p* < 0.05), especially CD4, which remained significantly lower than controls even at twelve months after transplantation (*p* = 0.011). B lymphocytes count was lower in the sixth month after HSCT in cases (*p* < 0.0001), but no difference was found at other follow-up time points. There was no difference in natural killer cells between the two groups (**Figure 2**). **Figure 3** shows the comparison of Ig series between the two groups. There was no difference in IgG levels at each follow-up time point. IgA recovered more slowly in the first and the twelfth month post-HSCT in CMVR cases. IgE was the sixth and the twelfth month while IgM presented a slower recovery trend in most follow-up months. We built linear regression models between the immunological cells and the CMVR/control groups. As shown in **Table 3**, linear regression results showed significant differences in T lymphocytes CD3, CD4, CD8, B lymphocytes, IgA and IgE between CMVR cases and controls. All these patients were tested for immune cells as part of routine post-transplant follow-up, one to five times. Considering the influence of disease grouping and follow-up time on the results, we further built two sets of mixed effect linear models to explore the interaction between disease grouping and follow-up time. The first set of model tested the cross level interaction between fixed effects and revealed an interaction between disease grouping and follow-up time in T lymphocytes CD3, CD8, IgA, IgE and IgM. The second set further confirmed the result after adjusting three variables with significant differences in constituent ratio including EBV co-infection, rituximab therapy and donor type (**Table 4**). **Figure 4** presents the overall trend of immune recovery of four T lymphocytes within one year post-HSCT.

**Table 3:** Mixed effect linear model of immunological indicators.

Variables	Linear regression				Mixed effect linear regression				
	Estimate	SE	*t*	*p* value	Estimate	SE	*t*	*p* value	Interaction *p* value
CD3 T lymphocytes	-1.36	0.21	-6.53	< 0.0001	-1.98	0.29	-6.93	< 0.0001	0.001
CD4 T lymphocytes	-1.23	0.19	-6.24	< 0.0001	-1.40	0.25	-5.63	< 0.0001	0.220
CD8 T lymphocytes	-1.41	0.22	-6.33	< 0.0001	-2.11	0.31	-6.81	< 0.0001	0.001
CD19 B lymphocytes	-0.88	0.27	-3.27	0.001	-0.74	0.34	-2.17	0.031	0.571
Nature killer cells	-0.03	0.11	-0.29	0.769	-0.14	0.19	-0.71	0.481	0.368
Immunoglobulin A	-0.11	0.03	-3.17	0.002	-0.04	0.06	-0.70	0.483	0.013
Immunoglobulin E	-0.51	0.24	-2.18	0.029	0.03	0.40	0.07	0.938	0.017
Immunoglobulin G	0.04	0.05	0.68	0.497	0.08	0.09	0.90	0.368	0.499
Immunoglobulin M	-0.002	0.05	-0.06	0.955	-0.22	0.07	-2.94	0.004	< 0.0001

**Table 4:** Mixed effect linear model of immunological indicators adjusted by variables with significant differences in constituent ratio shown in **Table 1**.

Variables	Linear regression^*^				Mixed effect linear regression^*^				
	Estimate	SE	*t*	*p* value	Estimate	SE	*t*	*p* value	Interaction *p* value
CD3 T lymphocytes	-1.41	0.21	-6.82	< 0.0001	-1.41	0.21	-6.71	< 0.0001	< 0.0001
CD4 T lymphocytes	-1.26	0.20	-6.36	< 0.0001	-1.27	0.20	-6.32	< 0.0001	0.0282
CD8 T lymphocytes	-1.45	0.22	-6.52	< 0.0001	-1.44	0.22	-6.56	< 0.0001	< 0.0001
CD19 B lymphocytes	-0.79	0.25	-3.06	0.0023	-0.44	0.26	-1.70	0.0896	0.2574
Nature killer cells	0.09	0.12	0.744	0.4565	0.06	0.16	0.36	0.7215	0.0710
Immunoglobulin A	-0.05	0.04	1.233	0.2179	-0.06	0.06	-0.93	0.3543	0.0206
Immunoglobulin E	-0.27	0.25	-1.07	0.2829	-0.29	0.34	-0.83	0.4053	0.0110
Immunoglobulin G	0.08	0.05	1.50	0.1346	0.09	0.07	1.26	0.2073	0.3781
Immunoglobulin M	0.02	0.05	0.37	0.7077	0.01	0.06	0.17	0.8682	0.0001

^*^Adjusted by EBV co-infection, rituximab therapy and donor type.

## Discussion

By dynamically observing the immune reconstitution of SAA HSCT recipients, we concluded that CMVR cases suffered more difficult T cell recovery soon after HSCT. A period of half a year after HSCT is the window for monitoring and predicting early CMVR infection. Most of the medical institutions that carry out HSCT conduct routine immune monitoring of transplant recipients, so it is practicable to initiate preemptive CMV therapy and timely cessation of treatment according to the hints and guidance provided by immunological parameters correlating with early CMVR. In our study population, low CD3, CD4, and CD8 T lymphocytes within six months post-HSCT, as well as low serum IgM, indicated an increased risk for CMVR. This result suggested that the delayed immune reconstitution cell population associated with CMVR was mainly T lymphocytes. The possible clinical factors influencing the immune reconstitution of SAA patients included EBV coinfection, rituximab administration, and alternative donors.

Several clinical studies have reported that CMVR occurs more frequently in human immunodeficiency virus (HIV)-infected patients with a low CD4 lymphocyte count. CD4 lymphocyte counts of 50 cells/µL or less have been clinically used to guide CMVR screening in HIV-positive and acquired immune deficiency syndrome patients, leading to rapid treatment and an improved prognosis for CMVR in these populations (Ford et al., 2013; Colby et al., 2014; Leenasirimakul et al., 2016). However, compared with HIV-infected patients with typical clinical features, CMVR in HIV-negative patients tends to present with atypical and variable fundus and clinical manifestations (Radwan et al., 2013; Lu et al., 2016). Consequently, the same CD4 cutoff value cannot necessarily be applied to CMVR in HIV-negative patients. Nor is it known whether any other immunological parameter might offer a similar predictive value in HIV-negative CMVR. Although our study has not confirmed the cutoff value for each type of immune cell, this study indicates that a low T lymphocyte count soon after HSCT correlates with a higher risk of late-onset CMVR. In addition, CD3 and CD8 T lymphocytes may have a predictive value similar to that of the CD4 T lymphocyte. Further efforts can be focused on exploring the optimal cutoff values for peripheral lymphocyte subsets in HSCT recipients to identify CMVR in larger prospective controlled trials.

Another interesting finding of this study was the potential predictive value of Ig series post-HSCT for CMVR. Except for no difference in the IgG level between the two groups, the other three presented a slower recovery trend in some follow-up time points in CMVR cases. Within six months post-HSCT, IgM especially recovered more slowly. While the field of CMV prevention and immunity has focused largely on T lymphocyte response, neutralizing antibodies may also play a role in controlling CMV infection post-HSCT for SAA patients (Pei et al., 2017; Haidar et al., 2020). Different from other report in haploidentical-HSCT for malignant disease (Yan et al., 2020),we observed a trend that CMVR cases exhibited lower serum IgM levels post-HSCT, although not every follow-up time point of analysis was significantly different. Different underlying disease, conditioning regimens and prophylaxis of GVHD may be related to different humoral immune recovery rules. Low serum IgM level may partially contribute to the incidence of CMVR. It should be noted that it is difficult to truly evaluate the serum IgG level in SAA HSCT recipients due to the intravenous administration of IgG according to the individual’s clinical condition.

Combined with clinical findings, we propose the following possible explanations for the delay in immune reconstitution in CMVR cases. First, CMVR occurred more often among the recipients from alternative donors. The risk of viral infections is higher in HSCT from alternative donors including haploidentical donors and unrelated donors than human leukocyte antigen-matched sibling donors due to their severely depressed T cell-mediated immune response (Atay et al., 2018). The addition of T cell-depleting agents, such as ATG, to the conditioning regimen and GVHD prophylaxis regimen in unrelated donors transplantation has been associated with a reduced incidence of GVHD but an increased risk of delayed immune reconstitution (Srinivasan et al., 2013; Ballen et al., 2016). In our study, there was a relatively low incidence of GVHD in CMVR cases, although acute (grade ≥ 2) and chronic GVHD has been identified as an independent risk factor for the development of CMVR after HSCT for primary immunodeficiency and hematological disorders (Crippa et al., 2001; Hiwarkar et al., 2014). We suspect that there was a low incidence of GVHD in our CMVR patients because all SAA recipients received ATG-based conditioning. Our group recently reported the pre-transplant risk factors for CMVR in SAA HSCT recipients (Zhang et al., 2022). We found that if a SAA patient ready for HSCT had a history of platelet refractoriness before transplantation or had only alternative donors available, the risk of CMVR after HSCT would increase. The current study used different cohort which included more research objects from different study period, although these were 117 patients including 24 CMVR and 93 non-CMVR patients overlapped in two studies. The two studies consistently identified alternative donors as a risk for CMVR in SAA HSCT recipients. Second, the percentage of EBV coinfections in the CMVR group was higher than that in the controls. Rituximab is an important agent in the preemptive treatment for EBV infection and therapy for EBV disease after HSCT. One main purpose of rituximab administration is to prevent EBV infection. As a result, we believe that the higher incidence of EBV infection and EBV disease in the CMVR patients is the main reason for the increased use of rituximab in the CMVR group. In addition, nearly half of the CMVR patients received rituximab therapy within six months after their HSCT, which may partly account for the delayed B lymphocyte recovery in the CMVR group at the time point of the sixth month after HSCT (shown as **Figure 2**). We noticed that B lymphocyte recovery was lower only at the sixth month after HSCT in CMVR cases, but no significant difference was found at other follow-up time points. Lack of helper function from lower number of CD4 T lymphocytes and the application of rituximab may be the main two reasons for lower B lymphocyte at the sixth month in CMVR cases. Finally, the CMVR patients in our study had a longer CMV duration, and higher CMV load compared to those patients who did not develop CMVR despite CMV infection. CMV can actively interfere with the reconstitution of protective immunity by infecting bone marrow stromal cells (Reddehase, 2016). The longer a CMV infection lasts, the longer the delay in the immune reconstruction.

In summary, this is a one-year follow-up observational study of immune reconstitution and viral monitoring coupled with regular fundus screening in SAA HSCT recipients. Our findings confirmed that alternative donor transplant was the major risk factor for the development of CMVR in SAA HSCT recipients. Delayed T lymphocyte recovery within six months post-transplantation was the key characteristics in CMVR patients which resulted in more severe CMV and EBV infection. The rituximab administration for EBV infection further delayed B lymphocyte reconstitution at the sixth month after HSCT. However, we cannot define the optimal cutoff values of T lymphocytes for the prediction of CMVR in SAA patients after HSCT without further confirmation from studies of larger sample size, as well as prospective studies. The identification of predictors of CMVR screening among SAA HSCT recipients could facilitate the risk stratification of patients for evaluation of new antiviral medications and other novel preventive and treatment strategies for CMV.

## Data Availability Statement

The original contributions presented in the study are included in the article/supplementary material. Further inquiries can be directed to the corresponding author.

## Ethics Statement

This study was approved by the institutional review board of Guangzhou First People’s Hospital (IRB B-2022-005-01) and was conducted in accordance with the Helsinki Declaration. Written informed consent from the patients/participants or patients/participants legal guardian/next of kin was not required to participate in this study in accordance with the national legislation and the institutional requirements.

## Author Contributions

WM was the major contributor in writing the manuscript. YZ designed the research. XC, XZ, and SW collected the patient data. LL analyzed the data and prepared the tables and figures for the manuscript. All authors reviewed the manuscript. All authors contributed to the article and approved the submitted version.

## Funding

This study was supported by the Guangdong Natural Science Foundation (2020A1515010215) and the Guangzhou Science and Technology Planning Project (202002030066).

## Conflict of Interest

The authors declare that the research was conducted in the absence of any commercial or financial relationships that could be construed as a potential conflict of interest.

## Publisher’s Note

All claims expressed in this article are solely those of the authors and do not necessarily represent those of their affiliated organizations, or those of the publisher, the editors and the reviewers. Any product that may be evaluated in this article, or claim that may be made by its manufacturer, is not guaranteed or endorsed by the publisher.
